# Preparation of Isotactic Polypropylene/Exfoliated MoS_2_ Nanocomposites via In Situ Intercalative Polymerization

**DOI:** 10.3390/polym9100490

**Published:** 2017-10-07

**Authors:** He-Xin Zhang, Xue-Quan Zhang, Keun-Byoung Yoon

**Affiliations:** 1 School of Chemistry and Chemical Engineering, Anhui University of Technology, Maanshan 243000, China; polyhx@ciac.ac.cn; 2 Department of Polymer Science and Engineering, Kyungpook National University, Daegu 702-701, Korea; 3 CAS Key Laboratory of Synthetic Rubber, Changchun Institute of Applied Chemistry, Chinese Academy of Science, Changchun 130022, China

**Keywords:** Ziegler–Natta, isotactic polypropylene, molybdenum disulfide, in situ polymerization, nanocomposites

## Abstract

In this research, a Ziegler–Natta catalyst intercalated MoS_2_ was synthesized through the intercalation of a Grignard reagent into MoS_2_ galleries, followed by the anchoring of TiCl_4_. During propylene polymerization, the intercalated MoS_2_ exfoliated in situ to form PP/exfoliated MoS_2_ (EMoS_2_) nanocomposites. The isotactic index values of the resultant PP/EMoS_2_ nanocomposites were as high as 99%, varying from 98.1% to 99.0%. It was found that the incorporation of the EMoS_2_ significantly improved the thermal stability and mechanical properties (tensile strength, modulus, and elongation at break) of PP. After introduction of EMoS_2_, the maximum increases in *T_d_*_5%_ and T*_dmax_* were 36.9 and 9.7 °C, respectively, relative to neat PP. After blending with commercial PP, the resultant nanocomposites increase in tensile strength and modulus up to 11.4% and 61.2% after 0.52 wt % EMoS_2_ loading. Thus, this work provides a new way to produce high-performance PP.

## 1. Introduction

Polyolefin is the most widely used materials because of its excellent combination of chemical and physical properties, as well as having low production cost, superior processability, and good recyclability. However, for advanced applications, it is necessary to improve the performance of polyolefin in terms of its properties such as stiffness and rigidity in forming nanocomposites. Thus, the study of polyolefin nanocomposites has attracted considerable attention, because of their high potential as materials with improved properties, such as mechanical and thermal stability, flame resistance, and thermal and electrical conductivities.

Molybdenum disulfide (MoS_2_) have enjoyed renewed interest owing to their crystallographic structure, which consists of covalently bonded S-Mo-S tri-layers in analogy to graphene [1,2,3,4,5]. According to the reports [6,7], MoS_2_ monolayers exhibit more higher mechanical properties (breaking strength: ~23 GPa; Young modulus: ~300 GPa) than chemically reduced graphene. Thus, MoS_2_ has a great potential to fabricate high-performance organic–inorganic polymer nanocomposites. Such as PVA, PS, PMMA, and chitosan have been successfully applied in nanocomposite fabrication with EMoS_2_ through a solution mixing method. After incorporation of EMoS_2_, the mechanical and thermal properties were significantly improved [8,9,10,11]. The uniform dispersion of EMoS_2_ in the polymer matrix can be achieved by solution mixing, which is the ideal strategy, wherein these polymers are dissolved in common organic solvents. However, the solution mixing process is difficult and uneconomical in the case of polyolefin, since these polymers are soluble only in solvents like xylene and trichlorobenzene above 120 °C. In our previous reports [12,13], the PE/EMoS_2_ nanocomposites were prepared through an in situ polymerization of ethylene using EMoS_2_ containing Ziegler–Natta catalyst. The resulting nanocomposites exhibited enhanced thermal and mechanical properties than neat PE. However, the catalyst preparation required complicated procedures, including a long lithiation of MoS_2_, violent exfoliation process, excessive amounts of water to remove lithium salt, a long vacuum freeze-drying process, and so on.

During the last decade, layered fillers (such as graphite and clay) have been successfully imbedded into polyolefins by in situ exfoliation method during olefin polymerization [14,15,16]. This approach is based on the intercalation of catalyst (Ziegler–Natta, metallocene, etc.) in the interlayers of the layered filler, followed by in situ polymerization. Due to the structural analogy to layered clay, a similar methodology can be applied to the synthesis of polyolefin/MoS_2_ nanocomposites.

Therefore, in this research, a Ziegler–Natta catalyst intercalated MoS_2_ was synthesized through the intercalation of a Grignard reagent into MoS_2_ galleries, followed by the anchoring of TiCl_4_. During the olefin polymerization, the bulk MoS_2_ layers are predicted to be exfoliated in situ and dispersed in the polymer matrix (no need additional exfoliation process), producing polyolefin/exfoliated MoS_2_ (EMoS_2_) nanocomposites directly.

## 2. Materials and Methods

### 2.1. Materials

Molybdenum disulfide (MoS_2_, ~6 μm), *n*-butylmagnesium chloride (BuMgCl, 2.0 M in THF), triethylaluminum (TEA, 1.0 M in hexane), diisobutylphthalate (DIBP), and titanium tetrachloride (TiCl_4_, >99%) were purchased from Sigma-Aldrich (Seoul, Korea) and used as received. Cyclohexylmethyldimethoxysilane (CHMDMS) donor and polymerization-grade propylene were received from Korea Petrochemical Ind. Co., Ltd., Ulsan, Korea. *n*-Hexane was distilled from sodium/benzophenone under N_2_ prior to use.

### 2.2. Preparation of MoS_2_-MgCl-Supported Ziegler–Natta Catalysts

An autoclave was charged with MoS_2_ (1 g), followed by addition of BuMgCl (20 mL), and was heated at 150 °C for 12 h under an argon atmosphere. Subsequently, the autoclave was cooled to room temperature, and the product was filtered and five times washed with anhydrous hexane. The resulting powder was suspended in *n*-hexane (200 mL) under ultrasonication for 30 min. Then DIBP (0.5 mL) was added, followed by dropwise addition of TiCl_4_ (10 mL) to the suspension at 0 °C. After that, the temperature was slowly increased to 80 °C (2 °C/min) and the suspension was stirred for 4 h. The mixture was filtered to remove the unreacted TiCl_4_, and the reactor was charged with fresh TiCl_4_ (20 mL). The reaction was complete after stirring for 4 h at 80 °C. After filtering, the solid product was washed several times with hot *n*-hexane (60 °C). Then, the obtained powdery catalyst was dried under vacuum at 60 °C for 3 h. The Mg and Ti contents of the powdery catalyst were determined by inductively coupled plasma atomic emission spectroscopy (ICP-AES; MoS_2_: 27.0 wt %, Mg: 9.6 wt %, Ti: 7.7 wt %). The catalyst was also synthesized without the addition of MoS_2_ for comparison. For this, a similar procedure was followed to prepare the catalyst without including MoS_2_ (Mg: 2.2 wt %, Ti: 13.9 wt %).

### 2.3. Propylene Polymerization

Propylene polymerization was performed in a three-neck glass reactor (300 mL). The reactor was thrice back-filled with N_2_ and charged with 100 mL distilled *n*-hexane. The reaction solution was stirred at 40 °C under 1 bar of propylene for 5 min, followed by addition of the co-catalyst (TEA) and CHMDMS donor. Subsequently, the catalyst was added into the reactor, and polymerization was started under a continuous feed of propylene (1 bar). After 2 h polymerization, 10 mL HCl-methanol solution (10%) was added to the suspension to terminate to polymerization. The mixture was poured into large quantity of methanol (500 mL) to precipitate the polymer. The product was collected by filtration and washed with methanol. Then, the product was dried under vacuum at 60 °C until a constant weight was achieved.

### 2.4. Characterization

The Mg and Ti contents of the catalyst were determined using inductively coupled plasma atomic emission spectroscopy (ICP-AES, PerkinElmer, Optima 7300DV, Houston, TX, USA). Scanning electron microscopy (SEM) images were recorded on a JEOL JSM-6380LV microscope (Hitachi, Japan). The morphology of the support and catalyst was studied by polarized optical microscopy (POM, ANA-006, Leitz, Wetzlar, Germany) using a CCD camera. X-ray diffraction patterns were obtained on a Philips X-Pert PRO MRD (Philips, Holland) diffractometer equipped with a Cu-*K*_α_ radiation source.

The polymer product was fractionated by extraction with boiling *n*-heptane for 8 h to determine its isotactic index (I.I.), calculated as the weight percentage of *n*-heptane-insoluble polymer. The melting temperature (*T*_m_) of the obtained polymer was determined using differential scanning calorimetry (DSC; DSC131evo, Setaram, Caluire, France) at a heating or cooling rate of 10 °C/min. The *T*_m_ was determined from the second scan. The decomposition temperature was determined under N_2_ atmosphere by thermogravimetric analysis (TGA; Setaram Labsys evo instruments) employing a programmed heating rate of 10 °C/min from 30 to 800 °C. The tensile mechanical properties of polymers were measured with a universal testing machine (Instron M4465, Instron Corp., Norwood, MA, USA). The resulting sample (1.5 g) was melt-blended with commercial PP (4.5 g) using a twin-screw mixer (HAAKE MiniLab Micro Compounder, Karlsruhe, Germany) at 190 °C and 100 rpm for 5 min. Five 5.0 × 75.0 × 1.0 mm^3^ samples were used for the tensile drawing experiment. The sample gauge length was 40.0 mm, and the crosshead speed was 50.0 mm/min.

## 3. Results and Discussion

**Scheme 1:**
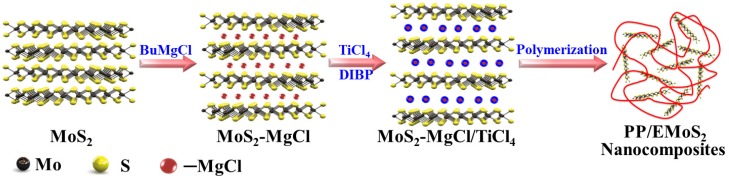
Preparation of the Ziegler–Natta catalyst intercalated MoS_2_ and PP/EMoS_2_ nanocomposites.

The preparation of the Ziegler–Natta catalyst intercalated MoS_2_ (MoS_2_-MgCl/TiCl_4_) and PP/EMoS_2_ nanocomposites with well-dispersed inorganic EMoS_2_ filler is illustrated in Scheme 1. In the first step, the Grignard reagent (BuMgCl) was intercalated into the MoS_2_ galleries and obtained MoS_2_-MgCl support, and then treated with excess TiCl_4_ to generate Mg/Ti catalyst species between MoS_2_ layers. During the polymerization process, the layered MoS_2_ will exfoliated in situ by the polymerization force arising from the propagation of polymer chain.

**Figure 1:**
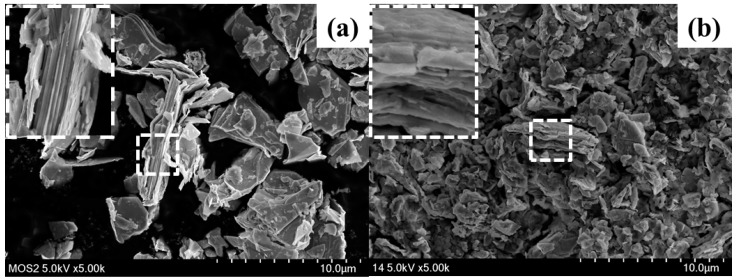
SEM images of (**a**) MoS_2_ and (**b**) Ziegler–Natta catalyst intercalated MoS_2_.

The morphologies of commercial MoS_2_ and Ziegler–Natta catalyst intercalated MoS_2_ were characterized by SEM and the images are given in Figure 1. It could be clearly seen that both MoS_2_ and the Ziegler–Natta catalyst intercalated MoS_2_ exhibited a sheet structure. Before intercalation, MoS_2_ contained a large number of tightly stacked thin single MoS_2_ layers. Interestingly, after intercalation of Ziegler–Natta catalyst into MoS_2_ galleries, the tightly assembled thin MoS_2_ layers became very loose, indicating the successful intercalation of catalyst into the interlayer spaces of MoS_2_.

**Figure 2:**
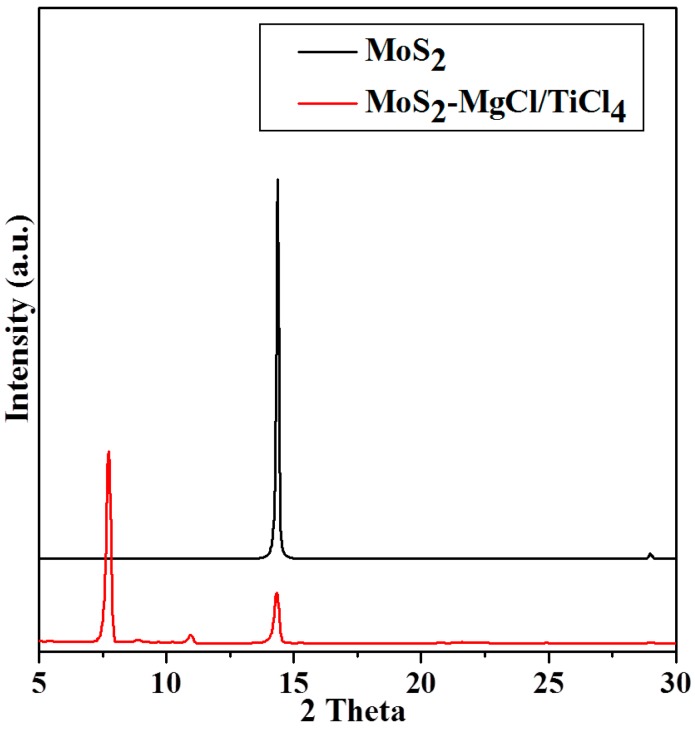
XRD patterns of MoS_2_ and MoS_2_-MgCl-supported Ziegler–Natta catalyst.

To confirm the successful catalyst intercalation into MoS_2_ galleries, XRD analysis of pristine MoS_2_ and the Ziegler–Natta catalyst intercalated MoS_2_ was conducted. As shown in Figure 2, an intense reflection at 2 theta = 14.3° (corresponding to an interlayer distance of 0.62 nm) was observed for commercial MoS_2_, attributable to the (002) plane. After treatment with the Grignard reagent and TiCl_4_, the above characteristic peak became very weak, and a new strong peak at 2 theta = 7.7° (corresponding to an interlayer distance of 1.2 nm) appeared. This clearly indicated that the expansion of interlayer space is due to the successful intercalation of the Ziegler–Natta catalyst into MoS_2_ galleries. Although the reflection peak at 2 theta = 14.3° could still be observed after intercalation, its intensity was drastically reduced compared to that of pristine MoS_2_.

The propylene polymerization behaviors of the catalysts in the absence and presence of MoS_2_ were evaluated after activation with the TEA co-catalyst. As shown in Table 1, the catalyst produced only trace amounts of PP in absence of MoS_2_, while the MoS_2_-MgCl-TiCl_4_ catalyst showed good activity towards propylene polymerization. By controlling the catalyst feed weight and [Al]/[Ti] ratio, PP/MoS_2_ nanocomposites with a MoS_2_ content of 0.4–2.1 wt % were obtained in this study. In the case of the MoS_2_-MgCl-TiCl_4_ catalyst, the isotactic index values of the resultant PP/EMoS_2_ nanocomposites were as high as 99%, varying from 98.1% to 99.0%.

**Figure 3:**
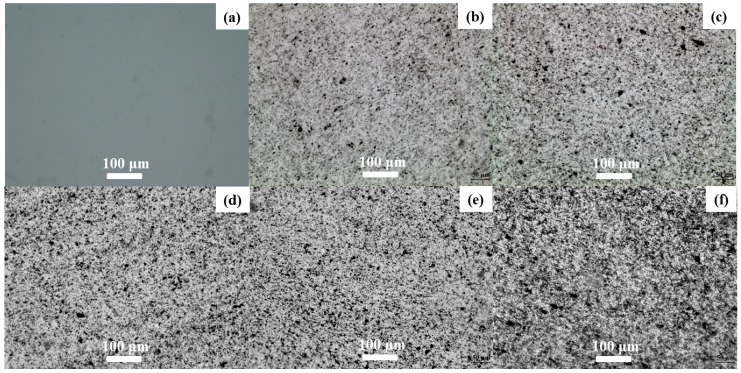
Optical micrographs of (**a**) PP and PP/EMoS_2_ nanocomposites with different EMoS_2_ contents: (**b**) 0.4 wt %; (**c**) 0.7 wt %; (**d**) 1.1 wt %; (**e**) 1.7 wt %; and (**f**) 2.1 wt %.

**Figure 4:**
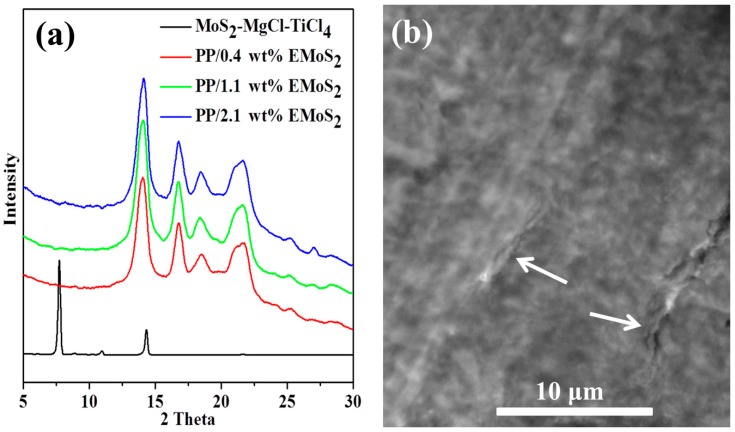
XRD spectra (**a**) of PP/EMoS_2_ nanocomposites and TEM image (**b**) of PP with 2.1 wt % EMoS_2_.

In order to investigate the dispersion of EMoS_2_ in the PP matrix, the resultant PP and PP/EMoS_2_ nanocomposites were hot-pressed into films. These films were observed in a transparent mode using an optical microscope; the obtained micrographs are listed in Figure 3. The MoS_2_ sheets were homogeneously dispersed in the PP matrix. Interestingly, although only 0.4 wt % of the EMoS_2_ filler was added, it could be clearly observed in the PP/EMoS_2_ nanocomposites. Larger amounts of the EMoS_2_ nanofiller could be observed in the PP matrix at increased EMoS_2_ feed ratios. Good dispersion of EMoS_2_ layers in the PP matrix without aggregation could also be observed. Dispersion of the latter filler in the PP/EMoS_2_ nanocomposites was further investigated by XRD and TEM, with the spectra shown in Figure 4. The PP/EMoS_2_ nanocomposites displayed diffraction peaks at 2 theta = 14.0°, 16.8°, 18.5°, 21.0°, and 21.8°, associated with the (110), (040), (130), (131), and (301) planes of PP, respectively. The peaks due to intercalation (7.7° and 11.0°) disappeared completely. The disappearance of these peaks is ascribed to the complete exfoliation of the intercalated EMoS_2_ by the chain propagation force of propylene polymerization. No conspicuous diffraction peaks were observed in addition to the ones of crystalline PP, indicating that no obvious stacking of EMoS_2_ sheets occurs in the PP/EMoS_2_ nanocomposites and that the stacked MoS_2_ sheets of the catalyst are completely exfoliated. Figure 4b is a TEM image of the PP with 2.1 wt % EMoS_2_ loading, showing that MoS_2_ is well exfoliated in PP matrix. Therefore, we expected the PP/EMoS_2_ nanocomposites to exhibit good thermal and mechanical properties.

**Figure 5:**
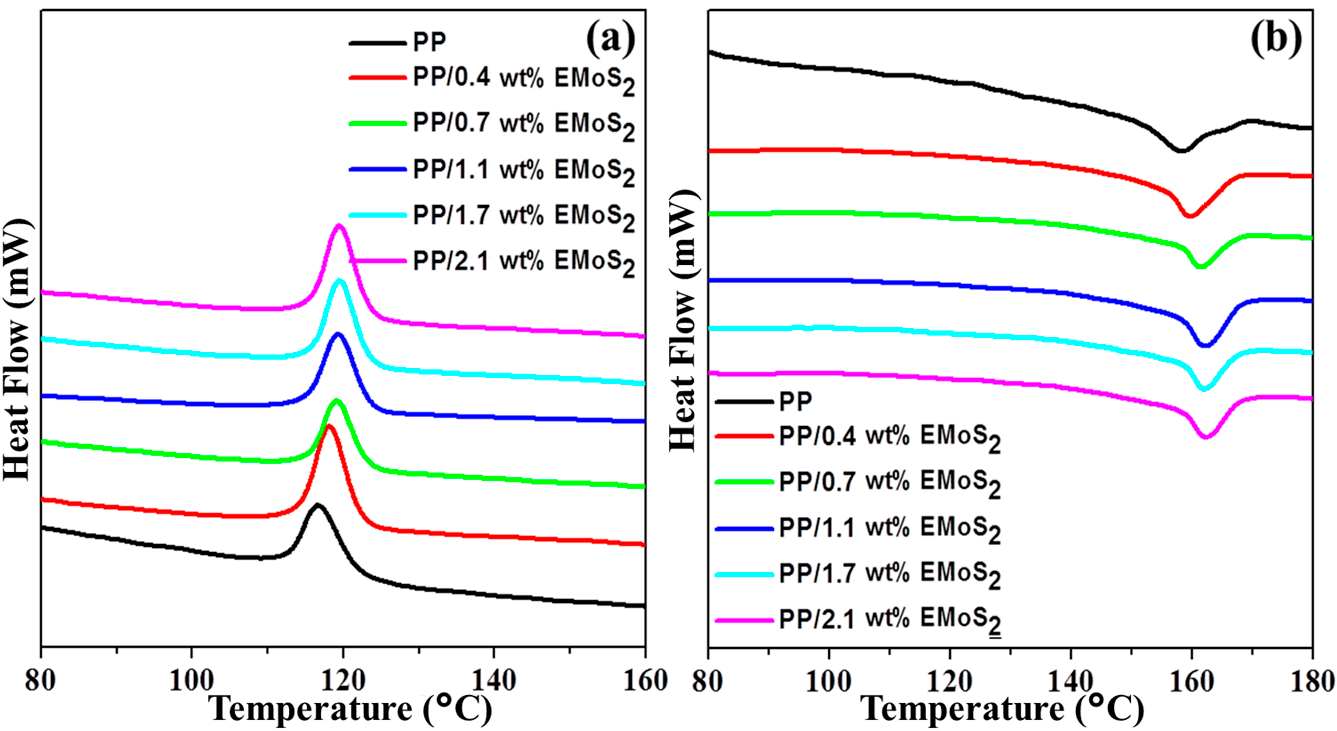
DSC (**a**) cooling and (**b**) heating curves of PP and PP/EMoS_2_ nanocomposites with different EMoS_2_ contents.

The effects of EMoS_2_ on the melting temperature, degree of crystallinity of PP are listed in Table 1 and the DSC curves are given in Figure 5. The degree of crystallinity was measured by means of DSC, using the ratio of fusion enthalpies of product to 100% crystalline PP. As shown in Table 1, the melting temperature (*T*_m_) of PP produced using the MoS_2_-free catalyst was 159.6 °C. Introduction of EMoS_2_ raised the *T_m_* of PP/EMoS_2_ nanocomposites, which could be ascribed to the restricted polymer chain motion (due to the interaction between the EMoS_2_ filler and the PP matrix) and the higher isotactic index of PP obtained using the MoS_2_-MgCl-TiCl_4_ catalyst [17]. Compared to neat PP, the non-isothermal crystallization peak temperature (*T*_c_) gradually increased with increasing EMoS_2_ content in PP/EMoS_2_ nanocomposites, which demonstrates that the above filler can act as a nucleating agent to induce PP crystallization. It is not surprising that Naffakh et al. [18] also reported a similar phenomenon for PP/MoS_2_ nanocomposites produced by melt mixing method.

**Figure 6:**
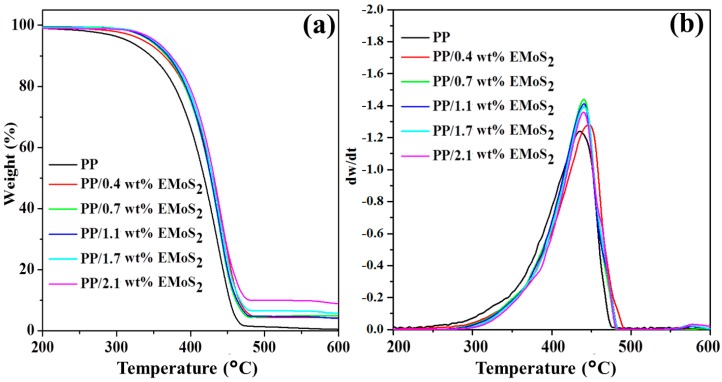
TGA (**a**) and DTG (**b**) curves of PP and PP/EMoS_2_ nanocomposites.

The thermal degradation of neat PP and PP/EMoS_2_ nanocomposites with different EMoS_2_ contents was confirmed by TGA under N_2_ atmosphere (Table 2 and Figure 6). Compared with neat PP, the thermal stability of PP was greatly improved by the incorporation of EMoS_2_ nanofillers. As shown in Figure 6, all TGA curves corresponded to a single degradation process and the thermal degradation curves were shifted to a higher-temperature region with increasing EMoS_2_ content, implying an improved thermal stability of PP. Table 2 shows that the degradation temperatures of all nanocomposites at 5 wt % loss (*T_d_*_5%_) are higher than that of pure PP. For 0.4, 1.1, and 2.1 wt % EMoS_2_ content in the PP/EMoS_2_ nanocomposites, the *T_d_*_5%_ increased by 21.6, 31.9, and 36.9 °C, respectively, relative to neat PP. With regard to 0.4 wt % EMoS_2_ loading, the temperature at the maximum degradation rate increased by 10 °C compared to neat PP. This significant enhancement of PP thermal stability following the incorporation of EMoS_2_ could be ascribed to the good dispersion of MoS_2_ in the PP matrix. This enhancement of polymer thermal stability upon incorporation of the EMoS_2_ filler has already been reported by both ourselves and other groups [8,9,10,12,13]. The char yield of neat PP is 0.7 wt % at 600 °C, while those of PP/EMoS_2_ nanocomposites are all higher, being in the range of 4.2–8.9 wt %. These results are not surprising, since Mo is a transition metal and can catalyze polymer char formation, while sulfur can improve flame retardancy [19,20]. Considering the above results, it is believable that the introduction of inorganic components into organic polymers, such as PP, can improve their thermal stabilities on the basis of the fact that EMoS_2_ fillers have good thermal stability due to the heat insulation effect of the EMoS_2_ layers and to the mass transport barrier to the volatile products generated during decomposition.

**Figure 7:**
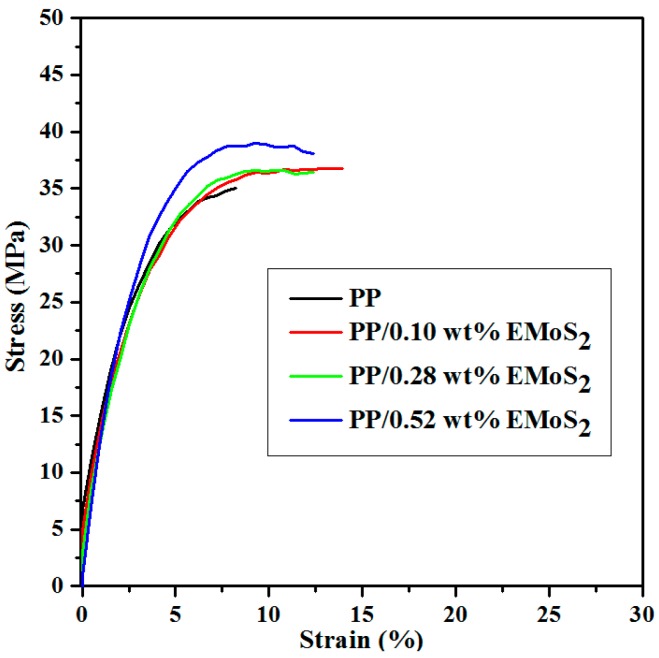
Stress–strain curves of PP and PP/EMoS_2_ nanocomposites.

The mechanical properties of PP/EMoS_2_ nanocomposites with 0.4, 1.1, and 2.1 wt % EMoS_2_ fillers were studied by melt-blending with commercial PP (1:3, mass ratio). The mechanical properties were summarized in Table 3 and the stress–strain curves were given in Figure 7. The tensile strength, modulus, and elongation at break values of PP/EMoS_2_ nanocomposites were significantly enhanced even at very low loadings of the EMoS_2_ nanofiller. At an EMoS_2_ loading of 0.10 wt %, the tensile modulus and tensile strength increased from 35.2 MPa and 850 MPa to 37.4 MPa and 960 MPa, respectively. When the EMoS_2_ loading increased to 0.52 wt %, the tensile strength of the PP/EMoS_2_ nanocomposites reached 39.2 MPa and 1370 MPa, while the elongation at break values also increased. The improved mechanical properties of PP/EMoS_2_ nanocomposites prepared by in situ polymerization method could be attributed to the good dispersion of EMoS_2_ fillers throughout the PP matrix. These results indicate that the PP/EMoS_2_ nanocomposites obtained by in situ polymerization using the MoS_2_-MgCl/TiCl_4_ catalyst provide a facile approach to efficient reinforcement of polyolefins.

## 4. Conclusions

A novel MoS_2_-MgCl-supported Ti-based Ziegler–Natta catalyst was successfully synthesized via intercalation of the latter catalyst into MoS_2_ galleries. PP/EMoS_2_ nanocomposites with well-dispersed EMoS_2_ nanofillers were successfully fabricated using an in situ exfoliation method during the propylene polymerization, displaying enhanced thermal stability compared to neat PP. After introduction of EMoS_2_, the maximum increases in *T_d_*_5%_ and *T_dmax_* were 36.9 and 9.7 °C, respectively, relative to neat PP. The resultant products were used as a masterbatch to reinforce the commercial PP through a melting blending method. The obtained PP/EMoS_2_ nanocomposites exhibited a significant improvement in mechanical properties, even at very small EMoS_2_ loadings. After blending with commercial PP, the resultant nanocomposites increased in tensile strength and modulus up to 11.4% and 61.2% after 0.52 wt % EMoS_2_ loading. Thus, this work provides a facile approach to the production of high-performance PP.

## Figures and Tables

**Table 1:** Results of propylene polymerization by the MoS_2_-MgCl-supported Ziegler–Natta catalyst.

Entry	Cat.	Cat. (mg)	[Al]/[Ti]	Activity (g/mol-Ti·h)	EMoS_2_ (wt %)	I.I. (%)	*T*_m_ (°C)	*T*_c_ (°C)	*X*_c_ (%)
1	BuMgCl/TiCl_4_	200	50	500	-	-	159.6	116.7	43.2
2	MoS_2_-MgCl-TiCl_4_	50	200	20,000	0.4	98.1	160.6	118.2	50.8
3		100	100	13,000	0.7	98.5	161.4	119.9	55.9
4		200	50	7600	1.1	98.1	161.5	119.3	61.2
5		300	34	5000	1.7	98.2	161.8	119.5	57.0
6		400	25	4000	2.1	99.0	162.6	119.5	58.9

Polymerization conditions: 100 mL *n*-hexane, [CHMDMS]/[Al] = 0.2, TEA co-catalyst, 2 h, 1 atm, 40 °C.

**Table 2:** Effect of EMoS_2_ content on the thermal stabilities of PP/EMoS_2_ nanocomposites.

EMoS_2_ Content (wt %)	*T_d_*_5%_ (°C)	*T_d__max_* (°C)	Char Yield (wt %)
-	315.1	435.3	0.7
0.4	336.7	445.0	4.2
0.7	345.2	440.6	4.9
1.1	347.0	441.5	4.2
1.7	350.5	438.3	5.8
2.1	352.0	440.3	8.9

**Table 3:** Mechanical properties of PP and PP/EMoS_2_ nanocomposites with various MoS_2_ contents.

Samples	EMoS_2_ Content (wt %)	Tensile Strength (MPa)	Modulus (MPa)	Elongation at Break (%)
Neat PP	-	35.2 ± 2	850 ± 80	7 ± 3
PP/EMoS_2_ nanocomposites	0.10	37.4 ± 2	960 ± 80	14 ± 4
	0.28	36.7 ± 2	1140 ± 100	13 ± 4
	0.52	39.2 ± 2	1370 ± 120	13 ± 4
